# 

**Published:** 1851-05

**Authors:** 


### CONTENTS

### OF NO. V.

---

### ORIGINAL COMMUNICATIONS.

### ESSAYS AND CASES.

ART. I.—An Anomalous Case of Horny Excrescences. By ASBURY EVANS, M. D., of Covington, Ky. - - 369

ART. II.—Pharmacy of the Greeks and Romans. Translated from the French of M. CAP, by W. H. COBB, M. D. 371

ART. III.—Practical Medicine. By THEODORE S. BELL, M. D., of Louisville, Ky. - - - - - - - - 384

### REVIEWS.

ART. IV.—A Practical Treatise on the most common Diseases of the South, exhibiting their peculiar nature, and the corresponding adaptation of Treatment. To which is added an Appendix, containing some miscellaneous matter. Also a Glossary explaining the meaning of the Technicalities, or medical phrases, used in this book. By THOMPSON MCGOWN, M. D., Graduate of Transylvania University, Member of the Lexington Medical Society, and a Practitioner of the South. "Is there no *balm* in Gilead? is there no *physician* there?" Why, then, may not all be healed, or profited? - - - - - - - - - 415

ART. V.—Ether and Chloroform: their employment in Surgery, Dentistry, Midwifery, Therapeutics, etc. By J. F. B. FLAGG, M. D., Surgeon Dentist, Member of the Rhode Island Medical Society.- - - - - - - 418

ART. VI.—The Medical Student' Guide in Extracting Teeth; with numerous cases in the surgical branch of Dentistry. With illustrations. By S. S. HORNOR, Practical Dentist. 430

ART. VII.—First Principles of Medicine. By ARCHIBALD BILLING, M. D., A. M., F. R. S. Second American, from the revised and improved fifth London Edition. - 432

ART. VIII.—Review of Chemistry for Students. Adapted to the courses as taught in the principal Medical Schools in the United States. By JOHN G. MURPHY, M. D. - - 433

ART. IX.—The American Journal of Science and the Arts. Conducted by Professors B. SILLIMAN, B. SILLIMAN, JR., and JAMES D. DANA, aided in the Departments of Chemistry and Physics by Dr. WOLCOTT GIBBS. New Haven, Conn. March and May numbers. - - - - - 435

Art. X.—Cases of Vesico-Vaginal Fistula, treated by operation. By George Hayward, M. D. - - - - 437
Art. XI.—An Address, delivered before the Suffolk District Medical Society, at its Second Anniversary Meeting, Boston, March, 1851. By Sam'l Parkman, M. D., M. M. S. S., one of the Surgeons of the Massachusetts General Hospital. - - - - - - - - - - - 439
Art. XII.—An Address to the Graduates of the Medical Department of the St. Louis University. By A. Litton, M. D., Professor of Chemistry and Pharmacy. - - - - 441
Valedictory Address before the Graduates of Washington University, Baltimore. By Professor T. E. Bond. - - 441
Valedictory Address to the Graduates of the Medical Department of the University of Pennsylvania. By W. E. Horner, M. D., Professor of Anatomy. - - - - - - 441
The progressive progress of Medicine in America: a valedictory address delivered before the graduating class at the first annual commencement of the N. Y. Medical Colleges By Horace Green, M. D., Prof. of Theory and Prac. Med. 442
Art. XIII.—Half-yearly Abstract of the Medical Sciences. Edited by W. H. Ranking, M. D., Cantab., assisted by W. W. A. Guy, M. D., George Day, M. D., Henry Ancell, M. D., and W. Kirkes, M. D. No. 12—July to December, 1850. - - - - - - - 446
Annual Report of the Louisville Marine Hospital. - - - 447
Report of the Diseases and their results, coincident with the Admissions and Discharges at the Louisville Marine Hospital, from March 1st, 1850, to March 1st, 1851. - - - 448

SELECTIONS FROM FOREIGN AND HOME JOURNALS.

On the Absorption of Alimentary Substances, and the Functions of the Lacteals. - - - - - - - - 450
On Iodognosis. - - - - - - - 451

ORIGINAL INTELLIGENCE.

The History of Medical Education, etc. - - - - - 454
Vaccination, Revaccination, Variola, etc. - - - - - 456
Professor Bartlett, - - - - - - - - - 457
Sinapisms, - - - - - - - - - - 458
Report of the Sanitary Commission of Massachusetts, 1850. - 458
Transactions of the Ohio State Medical Society, etc. - - 459
Obituary. - - - - - - - - - 460
Epidemic Influences, - - - - - - - 460

